# Dermatoskopie entzündlicher Hauterkrankungen

**DOI:** 10.1007/s00105-023-05122-9

**Published:** 2023-03-10

**Authors:** Julia Szebényi, Mária Légrádi, Csongor Németh, Marie Isolde Joura, Rolland Gyulai, Zsuzsanna Lengyel

**Affiliations:** 1 Klinik für Dermatologie, Venerologie und Onkodermatologie, Universität Pécs, Akác utca 1, 7632 Pécs, Ungarn; 2 Klinik für Dermatologie und Venerologie, Halmstad, Schweden; 3 Klinik für Dermatologie, Venerologie und Dermatoonkologie, Semmelweis Universität Budapest, Budapest, Ungarn

**Keywords:** Inflammoskopie, Psoriasis, Lichen planus, Dermatoskopische Merkmale, Spezifische Muster, Inflammoscopy, Psoriasis, Lichen planus, Dermoscopic characteristics, Specific signs

## Abstract

Das Dermatoskop wurde ursprünglich in der Dermatologie eingesetzt, um zwischen pigmentierten und nicht pigmentierten, gut- und bösartigen Tumoren zu unterscheiden. In den letzten 2 Jahrzehnten hat sich der Anwendungsbereich der Technik jedoch erweitert, und diese Untersuchungsmethode hat bei der Diagnose von Nichttumorerkrankungen, insbesondere von entzündlichen Hauterkrankungen, zunehmend an Bedeutung gewonnen. Bei der Beurteilung allgemeiner entzündlicher Hauterkrankungen wird empfohlen, nach der klinischen Untersuchung eine dermatoskopische Untersuchung durchzuführen. In der folgenden Zusammenfassung beschreiben die Autoren die dermatoskopischen Merkmale der einzelnen entzündlichen Hauterkrankungen. Zu den detaillierten Parametern gehören die Gefäßstruktur, die Farbe, die Schuppung, das Follikelmuster und das mit jeder Krankheit verbundene spezifische Muster.

## Abstract (en)

The dermoscope was initially used in dermatology to distinguish between pigmented and nonpigmented tumors, both benign and malignant. Over the last two decades, however, the spectrum of dermoscopy has broadened and its role in the diagnosis of nonneoplastic diseases, in particular inflammatory skin diseases, has become increasingly important. In the diagnosis of general and inflammatory skin diseases, it is recommended that dermoscopic evaluation should be performed after clinical examination. In the following summary, the dermoscopic features of the most common inflammatory skin diseases are described. Among the detailed parameters are the vascular structures, color, scaling, follicular findings, and specific signs associated with each disease.

In der klinischen Praxis werden neben der Beurteilung von pigmentierten und nicht pigmentierten Läsionen zunehmend auch entzündliche Hauterkrankungen (Inflammoskopie), Haarerkrankungen (Trichoskopie), Nagelerkrankungen (Onychoskopie) und bestimmte infektiöse Erkrankungen (Entomodermatoskopie) mithilfe der Dermatoskopie sichtbar gemacht, diagnostiziert und im Verlauf beobachtet.

Bei entzündlichen Hauterkrankungen wird die Inflammoskopie v. a. zur Bestätigung der klinischen Diagnose eingesetzt, kann aber ebenso den Verlauf einer Behandlung und die damit verbundenen und für eine gegebene Krankheit spezifischen strukturellen Veränderungen visualisieren und beobachten [1–4].

Für die dermatoskopische Untersuchung allgemeiner entzündlicher Hauterkrankungen wird empfohlen, die dermatoskopische Terminologie und die grundlegenden dermatoskopischen Parameter zu verwenden, wie sie in der 2019 veröffentlichten Konsenserklärung der International Dermoscopy Society (IDS) beschrieben sind. In dieser Publikation wird auf 5 grundlegende dermatoskopische Parameter zur Beurteilung allgemeiner Hauterkrankungen hingewiesen. Diese sind:

1. Morphologie und Anordnung der Gefäße,
2. Schuppen (Farbe und Verteilung),
3. follikuläre Strukturen,
4. weitere Strukturen (nicht vaskulär, nicht schuppend),
5. Hinweiszeichen, die für eine spezielle Erkrankung charakteristisch sind (wenn vorhanden) [1].

Die Verwendung dieser Terminologie bietet eine einheitliche, standardisierte Sprache.

Die dermatoskopischen Unterscheidungsmerkmale entzündlicher Hautkrankheiten sind oft weniger spezifisch als die von malignen und benignen Hauttumoren. Daher ist es wichtig, die Untersuchung allgemeiner entzündlicher Dermatosen mit der Beobachtung des klinischen Bildes unter Berücksichtigung möglicher Differenzialdiagnosen zu beginnen und anschließend die dermatoskopische Untersuchung durchzuführen („Zwei-Stufen-Modell“) [5].

Bei der dermatoskopischen Untersuchung wird die Läsion zunächst ohne Immersionsflüssigkeit analysiert, weil man dadurch die Schuppung und Kruste deutlicher sehen kann. Bei Anwendung einer Kontaktflüssigkeit sind diese möglicherweise weniger deutlich. Es ist wichtig, bei der Dermatoskopie nicht nur eine Hautläsion zu untersuchen, da dadurch weitere Charakteristika einer Hauterkrankung übersehen werden können (wie z. B. bei Skabies „Flugzeug mit Kondensstreifen“). Bei entzündlichen Dermatosen ist die Untersuchung der Blutgefäße wichtig. Das Dermatoskop sollte möglichst in kontaktfreier Form verwendet werden, da die Gefäße so am besten sichtbar sind.

Basierend auf der verfügbaren Literatur, heben wir in unserer Zusammenfassung die charakteristischen dermatoskopischen Merkmale bei den am häufigsten vorkommenden entzündlichen Hauterkrankungen hervor.

## Psoriasis

**Figure 1:**
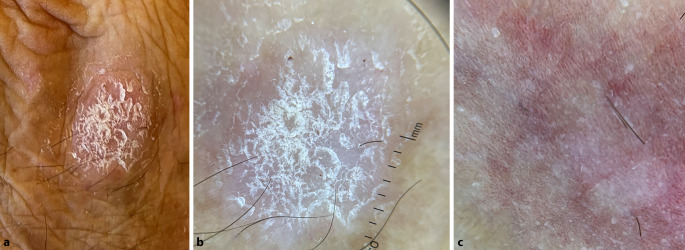

Das dermatoskopische Bild der Psoriasis ist durch gleichmäßig verteilte punktförmige Gefäße, gelegentlich globuläre Gefäße (per Definition ein Gefäß mit einem Durchmesser von mehr als 0,1 mm) und diffuse, weißliche Schuppung gekennzeichnet. Die punktförmige Gefäßstruktur entspricht histologisch den erweiterten Gefäßen in der Hautpapille [6]. Bei kräftiger Schuppung wird empfohlen, entweder die Schuppen vor der dermatoskopischen Untersuchung zu entfernen oder eine Kontaktflüssigkeit zu verwenden (Abb. 1).

Das Vorhandensein gleichmäßig verteilter, punktförmiger oder globulärer Gefäße ist spezifisch für die Psoriasis, aber diese Gefäßarten können auch bei anderen Krankheiten wie der lichenoiden Dermatose oder der Queyrat-Erythroplasie auftreten. Das Gefäßmuster der psoriatischen Hautsymptome in den unteren Extremitäten unterscheidet sich von anderen anatomischen Regionen. Klinische Studien haben bestätigt, dass in der Psoriasisplaque der unteren Extremitäten hauptsächlich regelmäßig angeordnete oder gruppierte globuläre Gefäße vorkommen, während eine punktförmige Gefäßstruktur seltener zu sehen ist. Die globuläre Erscheinung des Gefäßmusters lässt sich durch den höheren hydrostatischen Druck in den unteren Gliedmaßen und die beeinträchtigte Mikrozirkulation erklären. Ein ähnliches Muster kann bei einer Stauungsdermatitis oder Lipodermatosklerose beobachtet werden. Bei der videodermatoskopischen Untersuchung (70- bis 400fache Vergrößerung) der unteren Extremitäten haben diese Blutgefäße ein knäuelartiges Aussehen (dilatierte, spiralförmige Blutgefäße).

Das dermatoskopische Bild der unterschiedlichen Formen der Psoriasis ist sehr ähnlich. Der Grad der Schuppung hängt von der Lokalisation und der Psoriasisvariante ab. So ist die Schuppung bei der Psoriasis guttata und Psoriasis inversa in der Regel nicht ausgeprägt. Bei der palmaren, plantaren und auf der Kopfhaut lokalisierten Form ist das Vorhandensein einer ausgeprägten Hyperkeratose häufig.

Der Grad der Schuppung hängt von der Lokalisation und der Psoriasisvariante ab

Bei der Psoriasis capitis zeigt die dermatoskopische Untersuchung rote, punktförmige und globuläre Gefäße, strukturlose rote Areale, Siegelringgefäße und in der Epidermis den proximalen Teil des Haarschafts. Bei Vorhandensein der beiden letztgenannten Phänomene kann die Psoriasis als klinische Diagnose mit hoher Wahrscheinlichkeit gestellt werden und differenzialdiagnostisch von anderen nicht heilenden Alopezien unterschieden werden [7].

Die Merkmale der verschiedenen Formen der Psoriasis sind in Tab. 1 angeführt.

**Table 1:** 

	Gefäßstrukturen	Schuppung	Hintergrund	Andere
*Plaquepsoriasis*	Gleichmäßig verteilt punktförmig/globulär	Diffus verteilt, weiße Schuppung	Rot	–
*Psoriasis inversa*	Gleichmäßig verteilt punktförmig/globulär	±	Rot	–
*Psoriasis guttata*	Gleichmäßig verteilt punktförmig/globulär	Diffus verteilt, weiße Schuppung	Rot	–
*Psoriasis pustulosa*	Gleichmäßig verteilt punktförmig/globulär	±	Rot	Gelbe Kruste, weiße/gelbe Globuli, Pusteln lokalisieren sich nicht follikulär
*Erythrodermische Psoriasis*	Gleichmäßig verteilt punktförmig/globulär	Weiße Schuppung in Flecken	Rot	–
*Psoriasis capitis*	Rot, punktförmig, siegelringförmig, glomerulär	Perifollikuläre weiße Schuppung	Strukturlose rote Areale	Versteckte Haare
*Lichen ruber planus*	Punktförmig, globulär, linear (peripher)	Weiß, fleckig, dünn	Rot/lila	Wickham-Streifen, pigmentierte Strukturen (Punkte, Globuli), Rosetten, weißliche Struktur um die Komedoöffnung herum
*Ekzem (akut)*	Gruppiert, punktförmig, rot	Fokale weiße Schuppung	Gelbe flauschige Fläche	–
*Ekzem (chronisch)*	Gruppiert, punktförmig, rot	Weiße Schuppung (Lichenifikation)	Hautfarben oder rosa	–
*Asteatotisches Ekzem*	Gruppiert, punktförmig, rot	Weiße Schuppung (Bahnschienen-ähnliches Muster)	Hautfarben oder rosa	Gelblich-orangefarbener Flaum
*Nummuläres Ekzem*	Gruppiert, punktförmig, rot	Fokale gelblich-weiße Schuppung	Hautfarben oder rosa	Gelblich-orangefarbener Flaum
*Stasisekzem*	Gruppiert, glomerulär, globulär	Weiße Schuppung	Hautfarben oder rosa	–
*Seborrhoische Dermatitis (behaarte Kopfhaut)*	Spiralförmig, verzweigt, gebogen (kommaartig)	Gelblich-weiße Schuppung	Hautfarben oder rosa	–
*Atopische Dermatitis*	Fleckig, punktförmig	Fokale weiße Schuppung	Rosa	Gelbliche Kruste
*Pityriasis rosea*	Fleckig, punktförmig	Periphere weiße Schuppung (Collerette-Zeichen)	Diffuse/lokalisierte strukturlose gelblich-orange Bereiche	–
*Pityriasis rubra pilaris*	Linear, punktförmig	Nichtspezifische weiße Schuppung	Gelblich	Weiße keratotische Papel

**Figure 2:**
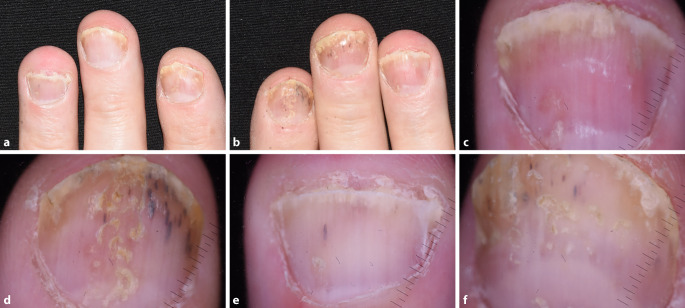

Fast die Hälfte der Patienten mit Psoriasis hat auch Symptome an den Nägeln. Die häufigsten Merkmale sind Splitterhämorrhagien, distale Onycholyse, lachsfarbene bzw. Ölflecken („salmon patches“/„oil drops“), subunguale Hyperkeratose und Grübchen („Tüpfelnägel“), von denen viele mit bloßem Auge sichtbar sind. Im Gegensatz dazu sind gewisse Abweichungen wie unscharfe (unregelmäßige breite weiße Lunula) und gesprenkelte Lunula, rote Punkte in der Lunula, längs verlaufende Nagelbetterytheme, erweiterte hyponychiale und erweiterte Nagelbettkapillaren oft nur mit dem Dermatoskop sichtbar. In Gegenwart von Nagelsymptomen ist die Wahrscheinlichkeit der Entwicklung einer Arthritis erhöht. In einer neueren Publikation wurde festgestellt, dass ein gleichzeitiges Vorhandensein einer subungualen Hyperkeratose und einer Onycholyse mit einer höheren Prävalenz einer Arthritis assoziiert ist ([8]; Abb. 2).

In mehreren Studien wurden die Veränderungen des dermatoskopischen Bildes nach einer antipsoriatischen Behandlung untersucht. Die Wirksamkeit der NB-UVB(Narrow-Band-Ultraviolett-B)-Therapie wurde mithilfe des Dermatoskops bei Patienten mit Psoriasissymptomen verfolgt. Das Ergebnis zeigte, dass psoriatische Hautläsionen, die globuläre Gefäße beinhalteten, weniger auf die Behandlung ansprachen als die Läsionen, in denen hauptsächlich punktförmige Gefäße zu sehen waren. Diese konnten eine relativ gute Erfolgsrate aufweisen [9].

Errichetti et al. verglichen mithilfe dermatoskopischer Bilddokumentation die Wirksamkeit einer topisch angewendeten Calcipotrien/Betamethasondipropionat-Behandlung (Schaum) bei Psoriasis-assoziierten Hautsymptomen von Studienbeginn bis zur 4. Woche. In der Studie wurden 105 kutane Läsionen bei 35 Patienten analysiert. Nach 4 Wochen wurde bei 13 Läsionen (12,4 %) keine oder nur eine minimale Besserung festgestellt, während bei 51 Hautsymptomen (48,6 %) eine teilweise und bei 41 Läsionen (39,0 %) eine komplette Remission zu verzeichnen war. Es wurde eine topische Therapieresistenz der Plaques in den unteren Gliedmaßen und den Streckseiten festgestellt. Bei Hautläsionen, die zu Beginn der Behandlung punktförmige Gefäße aufzeigten, konnte eine gute Wirksamkeit nachgewiesen werden, dagegen wurde bei Vorhandensein von globulären Gefäßen in Hautläsionen der unteren Gliedmaßen ein schlechter Therapieerfolg prognostiziert [10].

Heutzutage gibt es mehrere unterschiedliche Formen biologischer Therapien für die Behandlung der Psoriasis. Es ist bekannt, dass biologische Therapien eine Verbesserung der Mikrozirkulation herbeiführen und somit die in den psoriatischen Läsionen befindlichen Gefäßstrukturen verändern. Das Auftreten von hämorrhagischen Punkten in Hautläsionen (10fache Vergrößerung) ist ein früher prädiktiver Marker für ein gutes Ansprechen auf diese Behandlung. Wenn punktförmige Gefäße in den betroffenen Stellen persistieren bzw. erneut erscheinen, kann dies klinisch auf eine Stagnation oder auf eine wiederkehrende Läsion hinweisen [11, 12]. Micali et al. behandelten Patienten mit Adalimumab, Etanercept sowie Ustekinumab und überwachten den Kapillardurchmesser innerhalb einer Läsion mit einem Videodermatoskop (150fache Vergrößerung) vom Ausgangszeitpunkt bis zu Tag 15, 30 und 60. Bis zum 15. Tag wurde kein signifikanter Unterschied bei den Gefäßen festgestellt, aber am 30. Tag wurde eine Verringerung des Gefäßdurchmessers um 55,9 % (Adalimumab), 35,2 % (Etanercept) und 38,7 % (Ustekinumab) beobachtet. Bis zum 60. Tag wurde eine weitere Verringerung des Gefäßdurchmessers beobachtet (Adalimumab: 73,5 %, Etanercept: 49,7 %, Ustekinumab: 66,4 %) [13].

## Lichen planus

**Figure 3:**
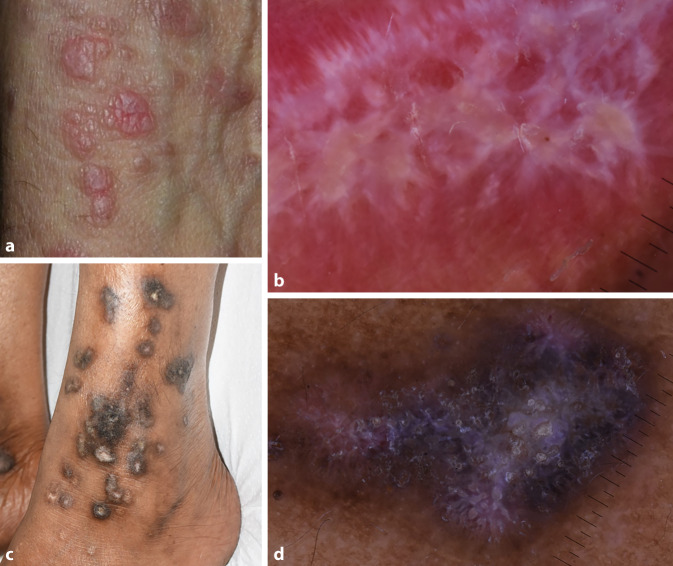

Das dermatoskopische Kennzeichen des Lichen planus sind die Wickham-Streifen, die histologisch mit einer Hypergranulose übereinstimmen (Abb. 3). Wickham-Streifen können in einer Vielzahl von morphologischen Mustern auftreten: linear, radial, anulär, baumartig verzweigt und auch als weiße Punkte („Sternenhimmel“). Sie sind in der Regel weiß, können aber bei dunkleren Hauttypen oder bei einer palmoplantaren Lokalisation gelb oder blau erscheinen. Es ist zu beachten, dass ein weißes Netzmuster auch bei anderen Hauterkrankungen (diskoider Lupus erythematodes, noduläre Skabies, Prurigo nodularis) festgestellt werden kann. Das Phänomen wurde als Pseudo-Wickham-Streifen beschrieben, dem wahrscheinlich eine Hautfibrose zugrunde liegt. Sie unterscheidet sich von den Wickham-Streifen durch das vaskuläre Muster der Läsion, bei der die Gefäßerweiterung stärker ausgeprägt ist.

Das dermatoskopische Kennzeichen des Lichen planus sind die Wickham-Streifen

Es ist wichtig zu betonen, dass das dermatoskopische Erscheinungsbild des Lichen planus von der Aktivität der Krankheit abhängt. Bei Papeln, die zu Beginn der Hautsymptome entdeckt werden, sind die Wickham-Streifen auf einem rötlichen Hintergrund nicht wirklich sichtbar. Bei kutanen Symptomen, die mit aktivem Lichen planus assoziiert sind, ist eine punktförmige, globuläre oder lineare vaskuläre Struktur, die sich hauptsächlich in der Peripherie befindet, ein gemeinsames Merkmal. Seltener ist die perifollikuläre oder diffuse Anordnung bzw. das Vorhandensein von weißen, gelblichen Punkten und pigmentierten Strukturen. Bei ausgeprägten Läsionen sind die Wickham-Streifen bereits deutlich sichtbar, und auch die peripheren Blutgefäße sind gut zu erkennen. Mit der Zeit verblassen die Wickham-Streifen, die auf den betroffenen Hautstellen zu sehen sind. Gleichzeitig erscheinen pigmentierte Strukturen auf der Läsion. Bei chronischem Lichen planus kann die pigmentierte Struktur das einzig sichtbare dermatoskopische Zeichen sein [5].

In einer Studie wurden verschiedene dermatoskopische Strukturen verglichen, die unter polarisiertem und nicht polarisiertem Licht sichtbar sind. Unter polarisiertem Licht waren eine „Vier-Knötchen-Rosette“ oder eine „Zwei-Knötchen-Rosette“, eine weiß leuchtende Linie, ein strukturloser, weißer Bereich und eine weißliche perifollikuläre Struktur deutlich sichtbar. Im nicht polarisierten Modus sind die Wickham-Streifen deutlicher zu erkennen, allerdings sind die oben genannten Merkmale undeutlich und schwer erkennbar. Die Autoren empfehlen daher, die dermatoskopische Untersuchung sowohl mit polarisiertem als auch mit nicht polarisiertem Licht durchzuführen [14].

Die klinischen Varianten des Lichen planus können unterschiedliche Muster aufweisen. Beim anulären Lichen planus erscheinen die Wickham-Streifen als anuläre Herde mit einer zentralen Abheilung. Oft sind sie mit Kapillaren oder Hyperpigmentierung assoziiert. Beim hypertrophen Lichen planus treten follikuläre Hornpfropfen, komedoartige Follikelöffnungen und gelbliche Areale auf. In diesem Fall sind die Wickham-Streifen nicht sichtbar, da sie unter der hyperkeratotischen Masse verborgen sind [15]. Beim porokeratotischen Lichen planus sind in dem Bereich, der von einem weißen hyperkeratotischen Rand umgeben ist, gepunktete Gefäße zu sehen, und in der Mitte sind bräunliche Pigmentflecken zu erkennen [16].

Beim Lichen planus pigmentosus werden meist schwarzbraune Punkte, globuläre Strukturen und selten Flecken beobachtet, die nicht in den Hautfalten auftreten. Die Punkte und globulären Strukturen können in einem den chinesischen Buchstaben ähnlichen Muster oder diffus angeordnet sein. Weitere Merkmale sind Teleangiektasie, ein pseudoretikuläres Pigmentnetzwerk oder eine zielscheibenartige Struktur (ein zentraler dunkler Punkt, umgeben von einem hypopigmentierten Halo) [17, 18].

## Ekzem

Das Ekzem ist eine entzündliche Erkrankung der Haut. Es gibt viele Typen von Ekzemen, die jedoch alle in der Histologie eine Spongiose aufweisen. Das dermatoskopische Erscheinungsbild ist durch eine Ansammlung roter, punkförmiger Blutgefäße und gelblich, manchmal gelblich-weißer Schuppen gekennzeichnet.

**Figure 4:**
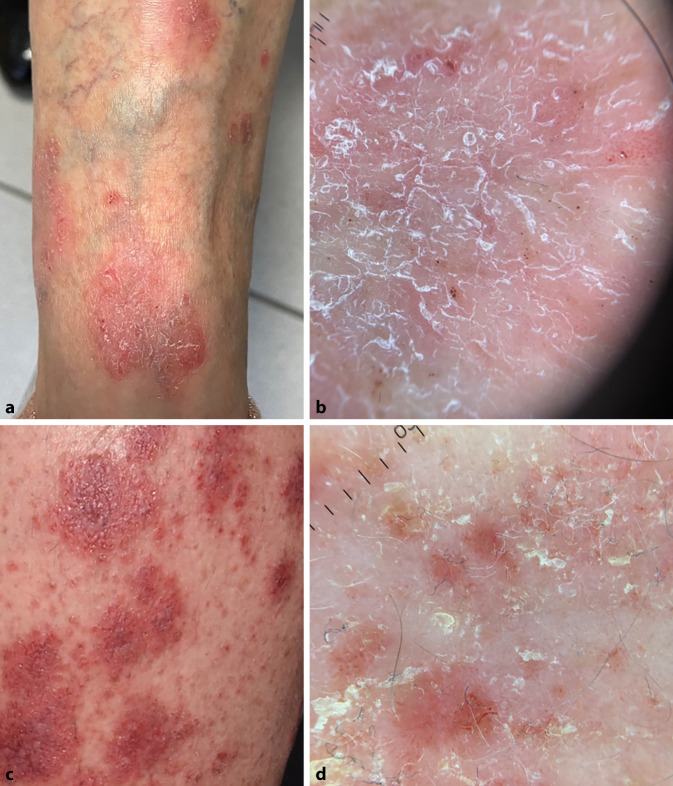

Die akute Phase der Erkrankung ist durch eine plaqueartig gelbliche Kruste („sero-crust“), Anhäufungen von punktförmigen Gefäßen sowie fokale gelbliche Schuppung gekennzeichnet (Abb. 4). Diese plaqueartig gelbliche Kruste („yellow clod sign“) stellt eine scharf begrenzte, münzförmige, erythematöse Läsion mit Sekret dar, die erstmals beim nummulären Ekzem beschrieben wurde [19]. Dadurch kann die Läsion gut von Psoriasis und Tinea corporis unterschieden werden.

In der chronischen Phase, bei Lichenifikation, sind weniger einheitliche, von einem weißen Halo umgebene, punktförmige Blutgefäße sowie weiße Schuppung charakteristisch. In der klinischen Praxis erleben wir häufig, dass sich diese Phasen überschneiden.

Bei einigen Ekzemarten können auch andere/besondere Veränderungen beobachtet werden. Beim asteatotischen Ekzem ist eine weiße Schuppung mit 2 freien Rändern, die Bahnschienen („rail-like“) nachahmt, üblich [5].

Das dermatoskopische Bild der Stauungsdermatitis ist durch die Anwesenheit von Schuppung und globulären oder glomerulären Gefäßen gekennzeichnet. Die glomerulären Gefäße entsprechen histopathologisch den erweiterten Kapillaren, die sich in den Hautpapillen und der papillären Dermis befinden.

**Figure 5:**
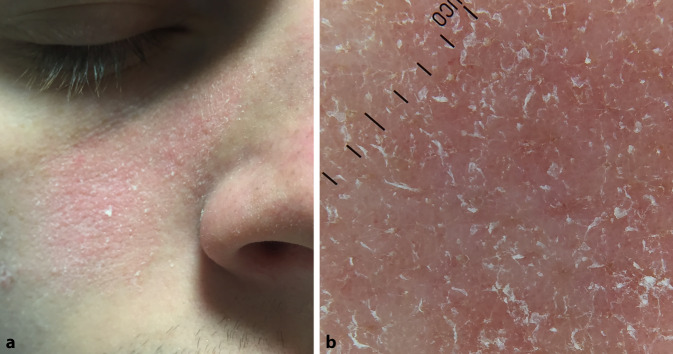

Bei der seborrhoischer Dermatitis sind die häufigsten dermatoskopischen Strukturen punktförmige, ektatische Blutgefäße, die von feiner gelblicher Schuppung begleitet werden (Abb. 5). Des Weiteren und seltener vorkommend sind auch follikuläre Hyperkeratosen, gelblich-orangefarbene Areale und lineare, verzweigte Blutgefäße zu erkennen.

Das trichoskopische Bild der seborrhoischen Dermatitis auf der Kopfhaut ist gekennzeichnet durch gelblich-weiße Schuppung, spiralförmige, verzweigte, kommaartige Gefäße [7]. Die Art und Form der Blutgefäße hilft uns, die seborrhoische Dermatitis der Kopfhaut von der Psoriasis capitis zu unterscheiden (punktförmig, globulär) ([20]; Tab. 2).

**Table 2:** 

	Hypertrophischer Lichen planus (HPL)	Prurigo nodularis	Psoriasis	Tinea corporis	Pityriasis rosea	Ekzem	Mycosis fungoides (Patch- und Plaque-Typ)
*Morphologie der Gefäße*	Punktförmig/linear	Punkförmig, glomerulär (erweiterte Gefäße)	Punktförmig	Punktförmig	Punktförmig	Punktförmig	Linear, manchmal punktförmig – Spermatozoon-ähnliche Strukturen
*Gefäßverteilung*	Peripherie	Peripherie	Gleichmäßig verteilt	Peripherie	Fleckenweise	Fleckenweise	Fleckenweise
*Schuppenfarbe*	–	–	Weiß	Weiß	Weiß	Weiß	Weiß
*Schuppenverteilung*	–	–	Diffus	Peripherie, mottenfraßähnliches Muster	Peripherie, dünn („Collerette“-Zeichen)	Fokal	Fleckenweise
*Weißer retikulärer Bereich*	Wickham-Streifen	Pseudo-Wickham-Streifen, weißer Sternenhimmel-ähnliches Muster	–	–	–	–	–
*Gelbe Serokruste („yellow sero-crust“)*		–	Weniger typisch (−/+)	Nicht typisch (−)	Typisch (+)	Typisch (+++)	Nicht typisch (−)
*Andere*	Komedoartige Öffnungen, Follikelverstopfung, gelbe Bereiche			Follikuläre Mikropusteln, abgebrochenes Haar			Orange-gelblicher Bereich in Flecken

Das dermatoskopische Merkmal der kutanen Manifestationen, die in den Hautfalten bzw. Beugen lokalisiert sind, ist die Anwesenheit von unregelmäßig angeordneten, linearen, verschwommenen Blutgefäßen. Bei diesem Typ wird jedoch die gelbliche Schuppung nicht beobachtet.

## Pityriasis rosea

**Figure 6:**
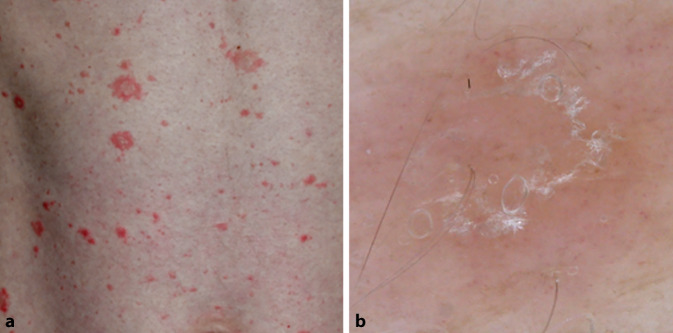

Ein charakteristisches Merkmal der Pityriasis rosea ist die weiße Schuppung am Rand der Läsion („Collerette“) (Abb. 6). Man sieht oft eine strukturlose, diffus verteilte oder lokalisierte, gelbliche Farbe im Hintergrund. Die Läsionen weisen meist punktförmige Gefäße auf, allerdings in Flecken und nicht in einer homogenen Anordnung wie bei der Psoriasis. Außerdem können rote und braune Globuli sowie Erythrozytenextravasate als helle Flecken vorhanden sein [5, 21].

## Pityriasis rubra pilaris

Pityriasis rubra pilaris gehört zur Gruppe der papulosquamösen Hauterkrankungen. Das dermatoskopische Erscheinungsbild der Hautsymptome ist durch das Vorhandensein weißer follikulärer Hornpfropfen und linear gepunkteter Blutgefäße auf einem gelblichen Hintergrund gekennzeichnet [5]. Bei der erythrodermischen Form der Pityriasis rubra pilaris ist eher ein oranger Farbton zu beobachten, und retikuläre Blutgefäße in nicht betroffenen Hautinseln sind charakteristisch. Bei der erythrodermischen Psoriasis sind die weißen Schuppen von einer gleichmäßigen Verteilung punktförmiger Gefäße begleitet, was zur Unterscheidung der beiden Erkrankungen beitragen kann.

## Rosazea

**Figure 7:**
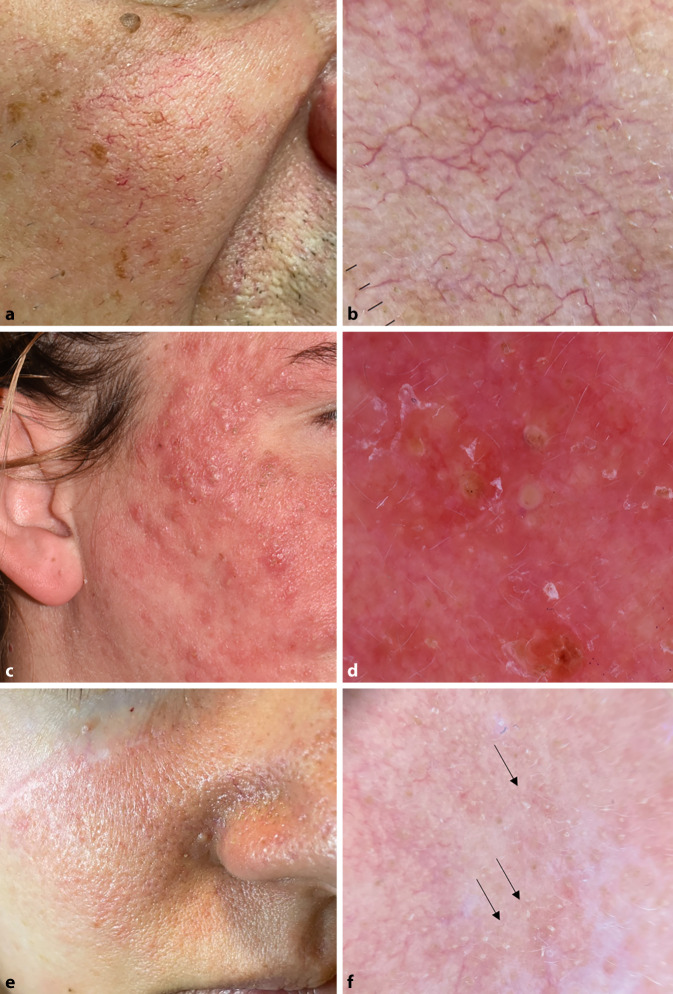

Rosazea ist ebenfalls eine chronisch entzündliche Hauterkrankung, die v. a. Wangen, Stirn, Kinn und Nase betrifft. Es gibt verschiedene Phänotypen (erythematoteleangiektatisch, papulopustulös, phymatös, okulär), die gleichzeitig auftreten bzw. von einem in den anderen Phänotyp übergehen können. Von den klinischen Formen der Rosazea ist das dermatoskopische Bild der Erythematoteleangiektasie die am meisten untersuchte Form. Sie ist durch das Vorhandensein eines regelmäßig verteilten, vollständigen bzw. unvollständigen polygonalen Netzes linearer Gefäße im Bereich der Läsion gekennzeichnet (Abb. 7a, b). Andere Strukturen wie Rosetten, weißlich-gelbliche Schuppen und erweiterte Follikel werden in der Literatur beschrieben, aber ihr Vorhandensein ist nicht spezifisch [22].

Eine kürzlich erschienene Publikation untersuchte die verschiedenen Rosazeatypen und stellte fest, dass beim papulopustulösen Subtyp das Gefäßmuster der erythematoteleangiektatischen Form ähnelt, jedoch weniger ausgeprägt ist und follikuläre Papeln sowie Pusteln vorhanden sind (Abb. 7c, d). Bei der phymatösen bzw. glandulär-hyperplastischen Rosazea war die Morphologie der Gefäße variabel (linear verzweigt, linear gepunktet), in einer retikulären Anordnung, und es waren follikuläre gelbe Globuli nachweisbar. Bei der granulomatösen Rosazea kann mithilfe der Dermatoskopie die Diagnose durch die Gegenwart fokaler, oranger, strukturloser Bereiche sowie das häufige Vorhandensein perifollikulärer oranger Farbe [22] unterstützt werden.

Bei der Differenzialdiagnose eines Gesichtserythems sollte auch an eine Demodikose gedacht werden. In diesem Fall kann man klinisch Rosazea-ähnliche Symptome mit follikulärer Hyperkeratose beobachten. Bei der Dermatoskopie wird am häufigsten eine follikuläre, weiße Masse („*Demodex*-Schwanz“) festgestellt, manchmal auch eine breite graue Farbe von 1–3 mm um die Follikelöffnung herum (*Demodex*-Follikelöffnung) ([22, 23]; Abb. 7e, f). Lineare Gefäße, die ein retikuläres Muster bilden, sind am häufigsten entlang des Erythems zu sehen.

## Schlussfolgerung

Mithilfe des Dermatoskops lässt sich eine Fülle von Informationen gewinnen, die zur Diagnose oder zur Differenzierung entzündlicher Hauterkrankungen beitragen können. Es sollte immer bedacht werden, dass dermatoskopische Parameter weniger spezifisch für eine bestimmte Krankheit als für einen Tumor sind, sodass bei Unsicherheit über die Diagnose eine histologische Entnahme empfohlen wird. Auf der Grundlage unserer bisherigen Erkenntnisse empfehlen wir bei entzündlichen Hauterkrankungen den regelmäßigen Einsatz der Dermatoskopie und die Verwendung einer standardisierten dermatoskopischen Terminologie, um ein einheitliches Verständnis gewährleisten zu können.

## Fazit für die Praxis

- Die Dermatoskopie ist eine nichtinvasive Untersuchungsmethode, die in der klinischen Praxis leicht anzuwenden ist.
- Sie hilft nicht nur bei der Diagnose von melanozytären und nichtmelanozytären Läsionen, sondern spielt auch eine wichtige Rolle bei der Erkennung von entzündlichen Hauterkrankungen.
- Bei entzündlichen Hauterkrankungen kann die Dermatoskopie verschiedene Strukturen aufzeigen. Zu den zu untersuchenden Merkmalen gehören die Gefäßstruktur, die Farbe, die Schuppung, das Follikelmuster und spezifische Muster bestimmter Krankheiten.
- Die dermatoskopische Untersuchung kann die Zahl der Biopsien, die einen invasiven Eingriff erfordern, verringern.
- Vor einer dermatoskopischen Untersuchung sollte immer eine klinische Untersuchung durchgeführt und eine Differenzialdiagnose gestellt werden.
- Untersuchen Sie immer mehrere Läsionen mit dem Dermatoskop und nicht nur eine.

## References

[1] Errichetti E, Zalaudek I, Kittler H (2020). Standardization of dermoscopic terminology and basic dermoscopic parameters to evaluate in general dermatology (non-neoplastic dermatoses): an expert consensus on behalf of the International Dermoscopy Society. Br J Dermatol.

[2] Errichetti E, Ankad BS, Sonthalia S (2020). Dermoscopy in general dermatology (non-neoplastic dermatoses) of skin of colour: a comparative retrospective study by the International Dermoscopy Society. Eur J Dermatol.

[3] Lallas A, Giacomel J, Argenziano G, García-García B, González-Fernández D, Zalaudek I, Vázquez-Lõpez F (2014). Dermoscopy in general dermatology: practical tips for the clinician. Br J Dermatol.

[4] Blum A, Fink C, Haenssle HA, Bosch S, Kittler H, Lallas A, Zalaudek I, Errichetti E (2020). Inflammoscopy: dermatoscopy for inflammatory, infiltrating and infectious dermatoses: indication and standardization of dermatoscopic terminology. Hautarzt.

[5] Errichetti E (2019). Dermoscopy of inflammatory dermatoses (inflammoscopy): an up-to-date overview. Dermatol Pract Concept.

[6] Ankad BS, Beergouder SL (2017). Dermoscopy of inflammatory conditions: the journey so far. EMJ Dermatol.

[7] Kibar M, Aktan Ş, Bilgin M (2015). Dermoscopic findings in scalp psoriasis and seborrheic dermatitis; two new signs; signet ring vessel and hidden hair. Indian J Dermatol.

[8] Yorulmaz A, Aksoy GG (2022). Dermoscopic features of nail psoriasis: revisited. Skin Appendage Disord.

[9] Errichetti E, Stinco G (2018). Clinical and dermoscopic response predictors in psoriatic patients undergoing narrowband ultraviolet B phototherapy: results from a prospective study. Int J Dermatol.

[10] Errichetti E, Croatto M, Arnoldo L, Stinco G (2020). Plaque-type psoriasis treated with calcipotriene plus betamethasone dipropionate aerosol foam: a prospective study on clinical and dermoscopic predictor factors in response achievement and retention. Dermatol Ther.

[11] Errichetti E (2020). Dermoscopy in monitoring and predicting therapeutic response in general dermatology (non-tumoral dermatoses): an up-to-date overview. Dermatol Ther.

[12] Lallas A, Argenziano G, Zalaudek I (2016). Dermoscopic hemorrhagic dots: an early predictor of response of psoriasis to biologic agents. Dermatol Pract Concept.

[13] Micali G, Lacarrubba F, Santagati C, Egan CG, Nasca MR, Musumeci ML (2016). Clinical, ultrasound, and videodermatoscopy monitoring of psoriatic patients following biological treatment. Skin Res Technol.

[14] Dash S, Behera B, Palit A, Sethy M, Nayak AK, Ayyanar P (2021). Dermoscopy of lichen planus under polarized vs. nonpolarized mode: a retrospective analysis of 14 patients. Clin Exp Dermatol.

[15] Hanumaiah B, Joseph J (2019). Role of dermoscopy in the diagnosis of hypertrophic lichen planus and prurigo nodularis. Indian J Dermatol.

[16] Dhanta A, Kansal NK, Durgapal P, Divyalakshmi C (2019). Porokeratotic lichen planus. J Dtsch Dermatol Ges.

[17] Devanda R, Kumari R, Rajesh NG (2022). Dermoscopy of lichen planus pigmentosus: a case series. J Am Acad Dermatol.

[18] Sonthalia S, Errichetti E, Kaliyadan F, Jha AK, Lallas A (2018). Dermoscopy of lichen planus pigmentosus in Indian patients—Pitfalls to avoid. Indian J Dermatol Venereol Leprol.

[19] Navarini AA, Feldmeyer L, Töndury B, Fritsche P, Kamarashev J, French LE, Braun RP (2011). The yellow clod sign. Arch Dermatol.

[20] Golińska J, Sar-Pomian M, Rudnicka L (2022). Diagnostic accuracy of trichoscopy in inflammatory scalp diseases: a systematic review. Dermatology.

[21] Lallas A, Kyrgidis A, Tzellos TG (2012). Accuracy of dermoscopic criteria for the diagnosis of psoriasis, dermatitis, lichen planus and pityriasis rosea. Br J Dermatol.

[22] Stefanou E, Gkentsidi T, Spyridis I (2022). Dermoscopic spectrum of rosacea. JEADV Clin Pract.

[23] Kara Y, Özden H (2021). Dermoscopic findings of rosacea and demodicosis. Indian J Dermatol.

